# A carbon dot-based clay nanocomposite for efficient heavy metal removal†

**DOI:** 10.1039/d3na00334e

**Published:** 2023-07-03

**Authors:** Khouloud Jlassi, Maryam Al Ejji, Abdelgalil Khalaf Ahmed, Hafsa Mutahir, Mostafa H. Sliem, Aboubakr M. Abdullah, Mohamed M. Chehimi, Igor Krupa

**Affiliations:** a Center for Advanced Materials, Qatar University P.O. Box 2713 Doha Qatar khouloud.jlassi@qu.edu.qa bakr@qu.edu.qa; b College of Arts and Sciences, Qatar University P.O. Box 2713 Doha Qatar; c Department of Chemical Engineering, College of Engineering, Qatar University Doha 2713 Qatar; d Université de Paris, ITODYS, UMR CNRS 7086 15 rue JA de Baïf 75013 Paris France mmchehimi@yahoo.fr

## Abstract

Carbon dots and their derivatives with fascinating photoluminescence properties have recently attracted tremendous scientific attention. This work describes the preparation of novel fluorescent bentonite clay (B), modified with carbon dot nanomaterials (CDs), and its usage as a lead removal platform. The CDs were prepared using a hydrothermal method from graphitic waste which served as the carbon source material. The as-obtained CDs were found to be fluorescent, being spherical in shape, positively charged, and smaller than 5 nm. Encouraged by their structure and photoluminescence features, they were used as surface modifiers to make fluorescent bentonite nanocomposites. Bentonite was used as a negatively charged model of aluminosilicate and reacted with the positively charged CDs. XRD, FTIR, XPS, and fluorescence analysis were used to characterize the prepared materials. The results indicate that the CDs intercalated inside the bentonite matrix were stable with excellent optical properties over time. They were finally used as an efficient hybrid platform for lead removal with a removal efficiency of 95% under light conditions, at room temperature, in an alkaline medium, and after only 10 min of reaction, compared to 70% under dark conditions. The pseudo-second-order kinetics and Langmuir isotherm models were better fitted to describe the adsorption process. The maximum adsorption capacity was equal to 400 mg g^−1^ toward Pb(ii) removal, at room temperature and pH = 8, under light conditions. To summarize, we have designed UV light stimuli responsive carbon dot-intercalated clay with high Pb(ii) adsorption capacity and long-term stability.

## Abstract 2

Carbon dots and their derivatives with fascinating photoluminescence properties have recently attracted tremendous scientific attention.

## Graphical abstract

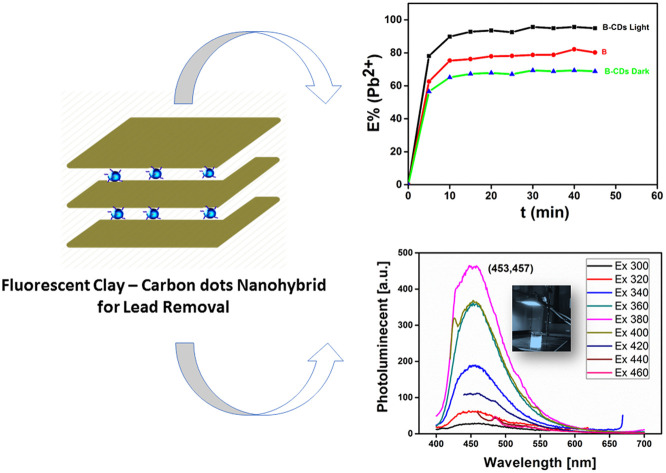

## Introduction

Photoluminescent hybrid nanomaterials recently exhibited a significant and unique role in scientific and technological developments and have been used in many fields, such as colorimetric sensing,^1^ light-emitting devices,^2^ fingerprint markers,^3^ photocatalytic hydrogen production,^4^ environmental remediation^5^ and a wide variety of other applications.^6^ In the last decade, CDs have emerged as exceptional photoluminescent nanomaterials due to their unique properties such as easy preparation,^7,8^ biocompatibility,^9^ easy functionalization,^10^ photostability,^11^ small size,^12^ and tunable emission.^13^ There are reports of the property-enhancing effects of doping CQDs with various dopants, including N, O, or S.^14^ As benign, inexpensive, and nontoxic fluorescent carbon nanomaterials, CDs could be an excellent candidate for an environment-friendly platform, especially for heavy metal removal.^15,16^ Using CDs, the pollutant removal mechanism typically happens through the fluorescence quenching of carbon quantum dots, whereby the interaction between the pollutants and functional groups of the fluorescent nanomaterial will change the energy states of the material and increase its recombination in the fluorescent nanomaterial; fluorescent quenching can also occur due to non-radiative electron transfer from an excited state in the fluorescent material to the orbitals of the contaminant.^17^ However, solid-state CD-based hybrid nanomaterials have raised much hope due to their higher stability than the suspension form of CDs.^1^ Therefore, some CDs cannot be directly used due to aggregation, which can lead to serious photoluminescence (PL) quenching.^1,2^ To overcome this problem, several organic and inorganic materials have been utilized to disperse the photoluminescent molecules (CDs) and maintain the PL properties of the desired solid state, such as in silica,^3^ starch,^4^ and clay nanotubes.^5^ As a low-toxicity, low-cost, and superior matrix material, bentonite clay has attracted significant attention as an inorganic filler of polymers.^6^ It is a layered aluminosilicate of substantial interest among 2D nanomaterials due to its abundance, low cost, high surface area, layered structure, anionic nature with excellent cation exchange capacity, and chemical reactivity (*e.g.*, silanization, arylation). Bentonite is employed in a variety of emergent domains and applications.^7,8^ Of relevance to this work, bentonite as a host inorganic material for developing photoluminescent systems has been previously described using photoluminescent cadmium selenide.^9^ However, dyes might undergo bleaching,^10^ whereas CDs are known to have aquatic toxicity,^23^ which is inappropriate for this work's targeted application. One could thus take advantage of the photostability of CDs and the biocompatibility of bentonite by designing photoluminescent clays. In previous years, bentonite clay was modified with carbon allotropes such as graphite,^11^ graphene,^12,13^ and multi-walled carbon nanotubes;^14,15^ the resultant hybrid materials were widely used in renewable energy, electronics, and environmental remediation applications. To this end, we combine the emerging photoluminescent CDs with bentonite to design a novel CD-intercalated layered aluminosilicate. To our knowledge, no study has been directed toward preparing natural layered clay intercalated with carbon dots. This is an essential step in the design of layered aluminosilicate/carbon nanomaterial as carbon dots have the unique feature of being fluorescent, which is not the case with CNTs and graphene. Hence, one could use CD-intercalated clay as a unique light-responsive material. In this work, based on blue emissive carbon dots prepared from graphitic waste which served as the carbon source and using a simple hydrothermal method at 120 °C, followed by ammonia treatment at 180 °C in a Teflon-lined autoclave, the final CDs were found to be co-doped with nitrogen and sulfur atoms, being spherical in shape, positively charged and smaller than 5 nm. Encouraged by their structure and photoluminescence features, they were used as surface modifiers to make fluorescent bentonite nanocomposites *via* a hydrothermal process. The association of CDs and bentonite in preparing the B–CDs composites is based on multifactor interactions, including the cation exchange reaction. Bentonite served as a host matrix to support, avoid the agglomeration and preserve the fluorescence properties of the as-prepared carbon dots. The resulting material is tested as a hybrid platform for lead removal from aqueous solutions.

## Experimental

### Chemicals and materials

Graphite waste was collected from Qatalum (Doha, Qatar). H_2_SO_4_, HNO_3_, ammonia, and bentonite nanoclay were purchased from Sigma-Aldrich (Munich, Germany).

### Synthesis of CDs and B–CDs

In order to obtain nitrogen-doped carbon dots (CDs), 4 g of graphite waste was initially ground and then dissolved in 100 mL of sulfuric and nitric acids (2/3 v/v) under stirring at 120 °C for 12 h. Then, the mixture was diluted, neutralized with ammonia, and then subjected to hydrothermal treatment in a Teflon-lined autoclave at 180 °C for 12 h, as previously described;^16^ the as-obtained CDs were introduced to a bentonite clay (B) suspension, to obtain the nanocomposite (B–CDs) through a cation exchange reaction. 1 g of B (negatively charged) was mixed with 20 wt% of CDs (positively charged), and the mixture was kept under mechanical stirring at room temperature for 24 h, centrifuged, washed and dried at 60 °C. The as-prepared material (B–CDs) was used hereafter as a hybrid platform for heavy metal removal and the heater for lead removal.

### Adsorption experiment

Adsorption processes for single-factor experiments used 0.01 g of B and B–CDs nanomaterials and 25 mL of lead solution (30 ppm, pH = 8) placed on an orbital shaker at 180 rpm and shaken between five and 45 min until equilibrium was reached. The inductively coupled plasma (ICP) technique was used to evaluate the remaining lead. The adsorption of B and B–CDs was followed by using the batch technique. All the adsorption experiments were repeated three times.

The removal efficiency of heavy metals was calculated using eqn (1):

1 % *R* = (*C*_0_ − *C*_e_/*C*_0_) × 100

where *C*_0_ and *C*_e_ represent the initial and remaining concentrations of heavy metals, respectively.

At equilibrium, the maximum adsorption capacity of lead was evaluated using the following equation (eqn (2)):

2 *Q*_e_ = (*C*_0_ − *C*_e_) × *V*/*m*

where *C*_0_ and *C*_e_ represent the initial and equilibrium concentration of lead in mg L^−1^, *V* is the total volume of solution in L, and *m* is the weight of the used adsorbent in mg.

To study the adsorption kinetics, the pseudo first- and second-order models were used, respectively, as described below (eqn(3) and (4)):

3 log(*q*_e_ − *q*_*t*_) = log *q*_e_ − *k*_1_*t*/2.303

4 1/(*q*_e_ − *q*_*t*_) = 1/*q*_e_ + *k*_2_*t*

Langmuir and Freundlich models (eqn (5) and (6)) were used to study the adsorption isotherms and evaluate the adsorbent's capacity toward heavy metals.

5 1/*q*_e_ = (1/*q*_m_*K*_L_)1/*C*_e_ + 1/*q*_m_

6 log *q*_e_ = log *K*_F_ + 1/*n* log *C*_e_

where *C*_e_, *q*_e_, *q*_m_, *K*_L_, and *K*_F_ represent the concentration of metal ions at equilibrium (mg L^−1^), the amount of metal ions (mg g^−1^), the adsorption capacity (mg g^−1^), and Langmuir and Freundlich equilibrium constants (L mg^−1^), respectively.

### Selectivity toward heavy metal removal

The prepared B–CDs and pristine bentonite clay were tested for the heavy metal removal application. To track their selectivity, they were immersed in three selected metal solutions, Zn(NO_3_)_2_, CuSO_4_, and CoSO_4_ (25 mL, 30 ppm), at room temperature, pH = 8, and under light conditions, and then washed several times with deionized water, and the supernatant was characterized using ICP to determine the metal removal rate.

### Characterization

The prepared materials, B, CDs, and B–CDs, were analyzed using a transmission electron microscope (TEM, Tecnai G220, FEI, Hillsboro, OR, USA), Thermo VG ESCALAB 250 instrument fitted with a monochromated Al Kα X-ray source, and by using an electron flood gun for charge compensation. The analyzer was operated at 40 and 100 eV pass energy for the narrow regions and survey spectra. SEM and N_2_ adsorption/desorption isotherms obtained using an ASAP 2020 Micromeritics surface area analyzer revealed qualitative information on the pore size distribution, pore volume, and specific surface area of B and B–CDs. The N_2_ adsorption/desorption isotherms obtained using an ASAP 2020 Micromeritics surface area analyzer revealed quantitative information on the pore size distribution, pore volume, and specific surface area of B and B–CDs. Elemental atomic concentrations were calculated from the XPS peak areas, and the corresponding Scofield sensitivity factors were corrected for the analyzer transmission work function. The X-ray diffraction pattern (XRD) was recorded on an X-ray diffractometer (X'Pert-Pro MPD, PANalytical Co., Almelo, Netherlands) using a Cu Kα X-ray source (*λ* = 1.540598 Å). The Fourier transform infrared spectra were characterized on a Thermo Nicolet Nexus 670 FTIR spectrometer (Thermo Scientific, Madison, WI, USA). The Raman spectra were recorded using a PerkinElmer Raman Station 400 spectrometer with an Ar^+^ ion laser at 780 nm. The spectra's baseline was corrected with a third-order polynomial and normalized to the intensity of the maximum peak. To investigate the optical properties, sample characterization was carried out with a Shimadzu UV-3600 spectrophotometer and FLS920 P fluorescence spectrometer (Edinburgh Instruments). The sample's lead (Pb(ii)) content was measured by ICP-MS using a NexIon 300d (PerkinElmer, USA).

## Results and discussion

### Preparation of CDs and B–CDs

The graphitic waste was collected from Qatar Petroleum and QATALUM Qatar Industries in Qatar, then ground using a cryomer to obtain fine powder (80 μm) of the previous waste. The crystalline structure of the collected graphite was investigated by X-ray structural analysis, which permits the determination of the structure's degree of order and the size of the crystallites. In this work, the XRD pattern typically shows one significant peak at 2*θ* = 25.7° and two weak peaks at 2*θ* = 43° and 2*θ* = 55°, corresponding respectively to (002), (100), and (004), showing significant crystalline structure of the graphite waste, as previously described^17,18^ (Fig. S1†). A formal application of the Scherrer equation suggests that the size of particles in this sample is about 17.9 Å. The FTIR spectra of both graphite waste and the as-prepared CDs are shown in Fig. S2;† for the graphite sample, no significant peak was observed, while the CDs reveal the presence of a different type of an oxygen-containing functional group; the band at 3441 cm^−1^ is assigned to the –OH group, the peaks at 1608 and 1763 cm^−1^ are due to the stretching vibration of C=O groups in the carboxylic moiety, the 1439 cm^−1^ peak is due to C–OH or C–H stretching vibrations and the 1128 cm^−1^ peak can be assigned to the C–O stretching vibration. The peaks situated around 1384 and 3153 cm^−1^ (the band beside the latter at 3441 cm^−1^ assigned to the –OH group; we can see only a minor deviation in the large band of O–H) are assigned to the C–N/C=N and N–H in-plane stretching vibrations of amine groups respectively,^19^ indicating the doping of nitrogen in CDs, and the two small peaks at 2918 and 2857 cm^−1^ are due to the vibration of –CH_2_ groups. The resulting CDs are rich in hydrophilic groups such as carboxyl and hydroxyl, formed from the graphite waste.

The new carbon dots (CDs) were prepared from graphitic waste *via* hydrothermal treatment in the presence of ammonia.

**Fig. 1:**
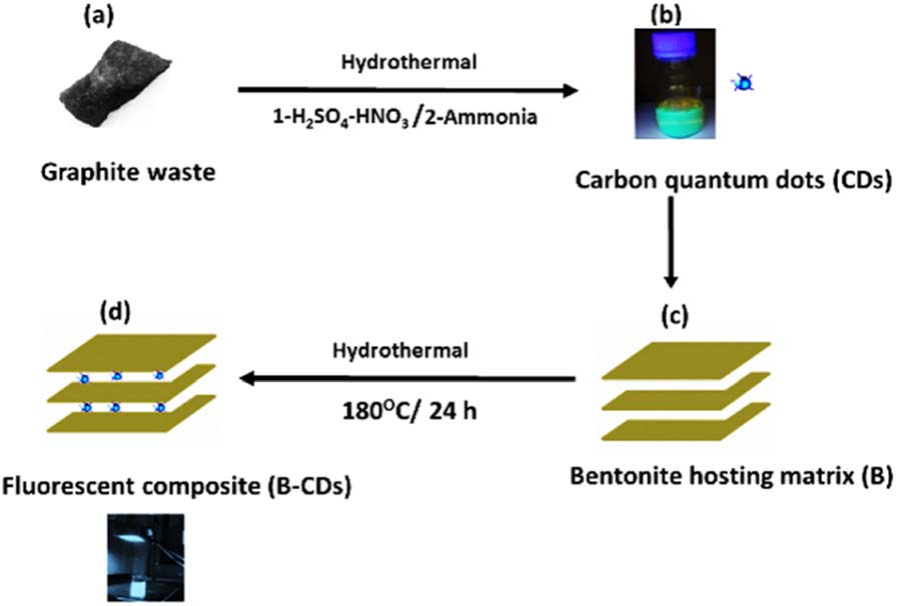
Upper panels (a) and (b) show the sequential steps of CD preparation; lower panels (c) and (d) show the surface modification of bentonite using the fluorescent CDs *via* a hydrothermal process to obtain fluorescent bentonite (B–CDs).

This process yields outstanding photoluminescent, monodisperse carbon dots with an average size smaller than 5 nm (Fig. 1a and b). The quantum yield of CDs was calculated by measuring the integrated PL intensity in an aqueous dispersion (refractive index *η* = 1.33) against quinine sulfate in 0.1 M H_2_SO_4_ (refractive index *η* = 1.33) as a standard one having a quantum yield of 54%, and quantum yield was estimated to be 28.7% (Table S1†).

CDs were used to make fluorescent bentonite clay *via* a hydrothermal process (Fig. 1b and c); the bentonite clay was used hereafter as a hybrid platform for heavy metal removal. The as-obtained CDs are nitrogen-doped and exhibit remarkable photoluminescence properties.

**Fig. 2:**
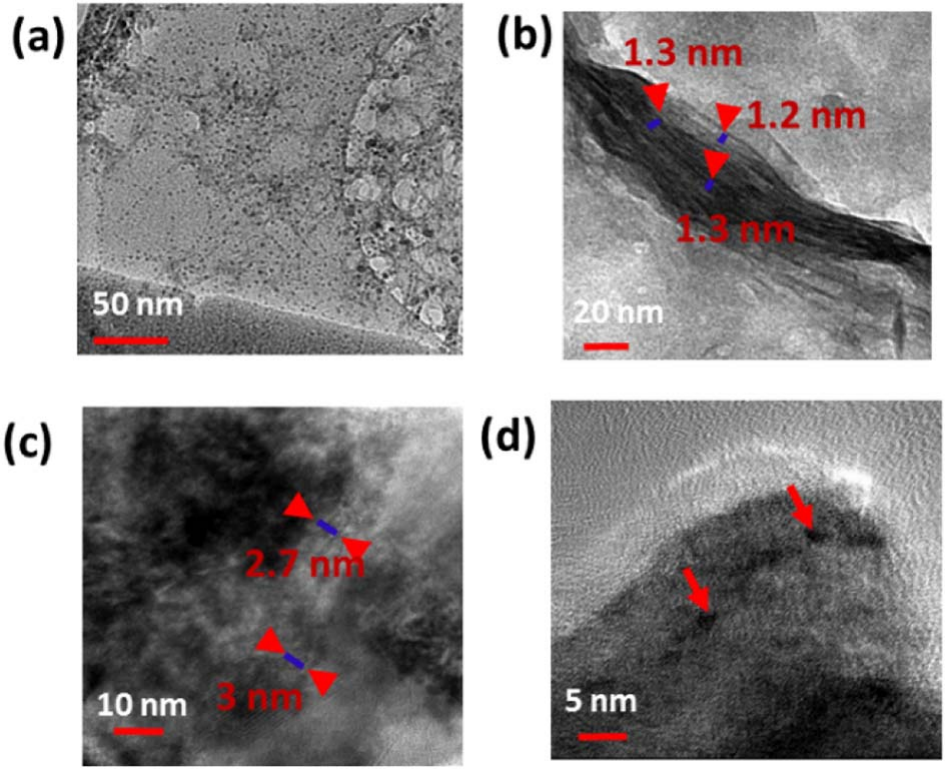
(a–d) TEM of B, CDs, and B–CDs at different magnifications.

**Fig. 3:**
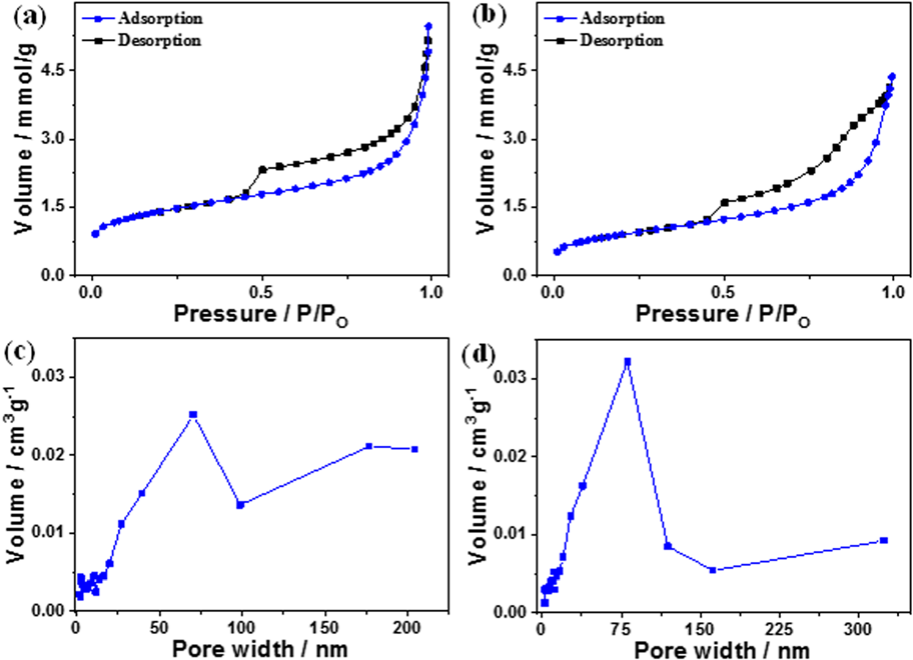
(a and b) N_2_ adsorption–desorption curves and (c and d) pore size distribution curves of B and B–CDs nanocomposite, respectively.

XPS was employed to investigate the electronic configuration and surface composition of B, CDs, and B–CDs, as the survey spectra permit to track the most critical changes in the surface composition of CDs and B surface after and before modification, and B–CDs exhibit intense N 1s and C 1s relative to Al 2p and Si 2p peaks from bentonite (Fig. 3a). TEM analysis of the carbon-coated grid by drop-casting, using a diluted solution of CDs, as shown in the images (Fig. 2a) revealed spherical and well-dispersed nitrogen-doped CDs, with an average diameter of 5 nm. From the corresponding X-ray diffraction (XRD) patterns of B and B–CDs (Fig. 2d) and applying the Bragg equation, the basal distance of pure bentonite was found to be equal to 1.3 nm after cation exchange reaction with the negatively charged CDs; the basal distance was found to increase up to 2.9 nm, indicating a successful cation exchange reaction, between the positively charged clay and CDs. The interlayer distance of B and B–CDs was also investigated using TEM images. The average distance was calculated through 2 selected regions and matched the distances deduced from XRD (Fig. 2b and c).

### Morphology

Fig. 2 shows the TEM of CDs, B, and B–CDs. Fig. 2a shows spherical CDs with an average size smaller than 10 nm. Some aggregation was observed due to some electrostatic interactions in the interfacial functional groups of the prepared CDs.^20,21^Fig. 2b shows the layered structure of bentonite clay; the basal distance from the three selected regions equals ∼1.2 nm; Fig. 2c and d show the intercalated clay layers in the presence of well-dispersed carbon dots; the average basal distance of bentonite clay was found to be equal to 2.8 nm. The diameter of spherical CDs is around 4.5 nm, and the size distribution is consistent with the Gaussian distribution (Fig. S3†) with a similar size obtained by the TEM image.

The BET and Langmuir techniques were used to measure the microscopic characteristics of B and B–CDs' surface area, while the BJH method was used to determine the pore volume and size distribution of B and B–CDs.

Fig. 3a and b show the N_2_ adsorption/desorption isotherms and pore size distribution of B and the B–CDs nanocomposite. According to the N_2_ adsorption/desorption curves, the B and B–CDs nanocomposite display a type (IV)-shaped isotherm; in the curve's center is an H1-type hysteresis loop, suggesting that the adsorbent is a typical mesoporous material.^22^

The pore size distribution of B and B–CDs is wide but non-uniform, as illustrated in Fig. 3c and d, with the bulk of pore diameters concentrated at around 55–75 nm. The Langmuir technique was used to calculate the specific surface area of B and B–CDs and the average pore size. The Langmuir adsorption values were 152.2 m^2^ g^−1^ and 100.0 m^2^ g^−1^ for the B and B–CDs nanocomposite, respectively.

The test results reveal that cation exchange modification with carbon dots slightly reduced the specific surface area, pore volume, and mean pore size of B to varied degrees. The following adsorption experiment shows that B–CDs can adsorb heavy metal ions due to the enhancement of specific pore size microscopic characteristics.

### Chemical structure

XPS analysis of the used materials before adsorption of Pb(ii) was conducted to investigate the surface composition of the B–CD nanocomposite and reference materials. Fig. 3a displays the survey spectra of B–CDs and CDs that confirm the presence of more intense C 1s and N 1s peaks for the B–CDs nanocomposite indicating modification of the clay by CDs.

**Fig. 4:**
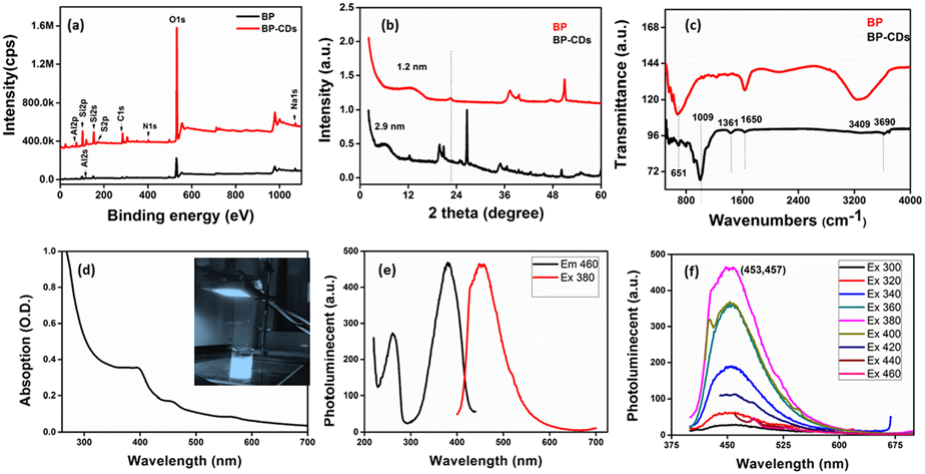
Upper panel: (a) XPS survey spectra of bentonite, CDs and their nanocomposite B–CDs; (b) XRD patterns of B and B–CDs nanohybrid. Middle panel: (c) FTIR spectra of B and B–CDs and (d) UV-Vis absorption spectrum. Lower panel: (e) PLE absorption spectrum and (f) excitation-dependent emission spectra of CDs at different excitation wavelengths.

FTIR spectra are shown in Fig. 4c for bentonite before and after modification with CDs. It is essential to note the decrease of OH band intensity, situated at 3413 and 1637 cm^−1^, reflecting the intercalation of the CDs with the clay surface. Moreover, the peaks at 651 and 1009 cm^−1^ could be associated with Si–O–Si, and Mg–O–Si vibrations;^23,24^ the peak at 1361 cm^−1^ for the B–CDs material is associated with C–O bond vibration, and the one at 1457 cm^−1^ is assigned to C–N bonds. Moreover, C=C/C=O bonds could be detected through the band centered at 1650 cm^−1^, while the band at 3409 cm^−1^ indicates the presence of O–H and N–H groups in the B–CDs material.^25^ The XRD patterns of B, and B–CDs are shown in Fig. 3b. The typical diffraction peaks related to bentonite shift to smaller angles after the modification with CDs, as concluded from the Bragg equation. The basal distance of bentonite clay shifted from 1.2 to 2.9 nm for B–CDs respectively, in the same range of the basal distances concluded from TEM images (Fig. 2b). In contrast, the XRD pattern of B–CDs recorded in some regions shows some additional diffraction peaks along with those for bentonite. The peaks centered at 2*θ* = 27° are attributed to the facets of graphitic-like carbon. Fig. S4† shows the Raman spectra for graphite and CDs. It is worth mentioning that the light excitation source was fixed at 785 nm, as it is one of the most accurate approaches to investigate the disorder in sp^2^ carbon materials. The Raman spectrum for CDs shows the typical G-band at 1590 cm^−1^ attributed to the sp^2^ C atom bonds and a D-band at 1330 cm^−1^ corresponding to the C atoms disorder at the edges. Reportedly, the lessening of the intensity of the D-band peak is owing to the increased graphitization and crystallinity of the as-synthesized material. Meanwhile, a more substantial increase of the G-band intensity than the disordered D-band assumes the augmented graphitization for the CDs. Additionally, the ratio between *I*_D_ and *I*_G_ values, which elucidates the disorder in the graphene structure, is around 1.3, confirming the uniformity of the as-synthesized material. For CDs, the G-band appears at 1590 cm^−1^ and the D-band at about 1360 cm^−1^. The *I*_D_/*I*_G_ ratio represents the disorder in graphene structure. For CDs it is 1.36, while for graphite it is around 1.06, suggesting that adding nitrogen atoms into the exterior conjugated carbon backbone results in more disordered structures; blue shifts of D and G bands can be seen, consistent with the previous results.^26,27^

These TEM and XRD results account for the intercalation of CDs into the bentonite matrix. The UV-Vis absorption spectrum of the B–CDs nanocomposite (Fig. 4d) displays two absorption peaks centered at 380 and 450 nm, assigned to π → π* transition and n → π* transition, similar to previously reported carbon dot-based materials.^16,28^ The B–CDs excited under 340 nm UV light show a blue color (Fig. 4d). The photoluminescence properties of the nanocomposites were evaluated with the help of photoluminescence spectroscopy. The emission spectra (Fig. 4f) of the B–CDs show the increase in the PL intensity as the excitation wavelength increases from 300 to 460 nm; the emissions were significantly red-shifted, common in carbon dot-based materials.^29^ The prepared nanocomposite emits the maximum intensity (454 nm) when excited at 380 nm (Fig. 4e); the PL spectrum was fitted to two Gaussian peaks, called intrinsic and extrinsic emission peaks (P1 and P2), as shown in Fig. S10.† The P1 peak represents core emission (FWHM = 49 nm) and a broad more substantial P2 peak (FWHM = 55 nm) that originates from defects on the core edges. This phenomenon is associated with the quantum core effect due to the conjugated domains in the carbon dot core, a defective or surface state PL due to doping with C–N/S/OH groups, and PL due to fluorophores and crosslink-enhanced PL emission.^30^

### Application to the removal of Pb(ii) from water

The removal of Pb(ii) from the aqueous solution was evaluated using pure bentonite (B) as well as the prepared nanohybrid material (B–CDs) under dark and light conditions. The removal of the metal ions was assessed using the ICP analysis technique. The selectivity test was performed in the presence of Cu^2+^, Zn^2+^, and Co^2+^ at pH 8.0, and the relative removal efficiency equals 48, 33, 66, and 95%, respectively (Fig. S5†).

**Fig. 5:**
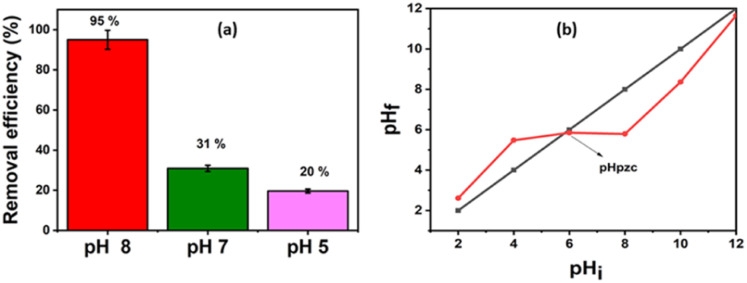
(a) Trends of the removal efficiency percentages for the lead at different pH; (b) point of zero charge of NCS/SA (*T* = 25 °C; *m*_ads_ = 0.015 g and *V* = 50 mL).

The adsorption performance of Pb(ii), using B and the B–CDs nanohybrid, was also investigated *versus* pH and contact time. B and B–CDs were immersed in a 30 ppm Pb(ii) solution for up to 45 min. The remaining concentration of Pb(ii) was detected using the ICP analysis technique; as shown in Fig. 5a, the removal efficiency percentage of Pb(ii) was found to increase in alkaline solution, and the highest Pb(ii) removal efficiency percentage (*E* = 95%) was noted in alkaline solution at pH = 8 at room temperature (Fig. 5b), using B–CDs under the light stimulus, compared to 81% and 70%, respectively, only in the presence of unmodified B and B–CDs under dark conditions; this could be explained using the point of zero charge (PZC) for B–CDs using the drift method, which involves adding the adsorbent to solutions of a range of increasing pH. The initial pH is set, and the final pH is measured after 24 h of mechanical stirring. The intersection of the initial pH *vs.* final pH graph gives the value of PZC. For the case of B–CDs, the PZC was obtained as 6, which can explain why the clay's highest performance is observed at pH 8. Below the point of zero charge, the material has an *overall positive net charge*; however, when in a solution with a pH above the PZC value, the adsorbent material has an overall negative charge.^31^ Because of this negative charge, the cation exchange capacity of B–CDs increases, which enables better lead exchange. Moreover, the collected dried adsorbents (B–CDs–Pb) were chemically and thermally stable, as shown in Fig. S7 and S8.†

**Fig. 6:**
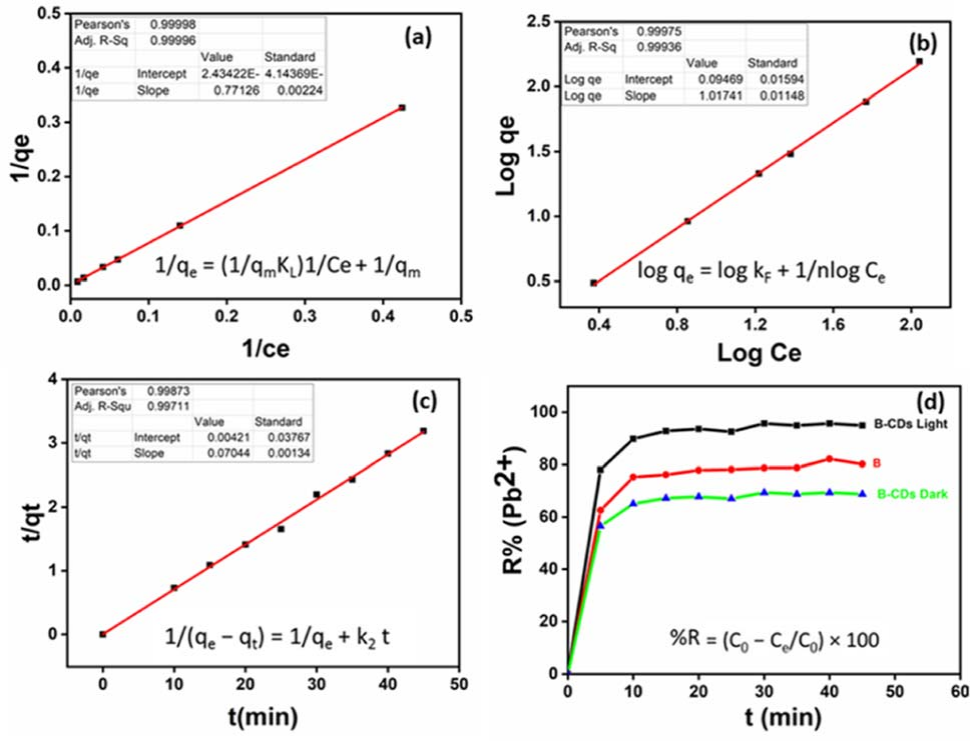
(a) The relevant results of Langmuir models, (b) Freundlich models, and (c) the pseudo-second-order model for Pb(ii) adsorption using B–CDs under light conditions. (d) Trends of the extraction efficiency percentages for cadmium using B and B–CDs under light at pH = 8.

After UV light irradiation (365 nm) for 30 min, the removal of Pb(ii) was almost complete (95%) compared to that under the dark conditions (70%). From the Fig. 6d plots, the *E*% of Pb(ii) *versus* reaction time shows that photo-adsorption is ultra-fast and efficient. Such a high adsorption activity of B–CDs may be attributed to the O, N, and S dopants of the prepared carbon dots, which reduce the band gap energy,^32^ delay the electron–hole recombination, and enhance the light absorption of B–CDs, resulting in an increased electron density and improved attraction of the positively charged Pb(ii).^33,34^ Nevertheless, the mechanism has not been fully explained in the literature.

To test the regeneration capacity of the B–CDs material *via* Pb(ii) removal, the reusability of the used adsorbent was investigated by performing five successive adsorption–desorption cycles; a procedure was carried out by desorbing the lead ions from B–CDs using acid treatment.^35^ The clay was centrifuged in 0.1 M nitric acid for 30 min and then used for the adsorption study as done initially. This cycle was repeated four times, and the removal efficiency percentage of each cycle has been calculated and is displayed in Fig. S6.† The adsorption performance of the clay decreases significantly after the first cycle and decreases gradually in the consecutive cycles.

The adsorption isotherm is applied to estimate the adsorption capacity of an adsorbent. As done in most previous studies over the last two decades, we have used the classical pseudo-first-order and pseudo-second-order models from among the adsorption kinetic models (eqn (3) and (4)) to study the variations in adsorption with time.^36,37^ These two models have been applied to various adsorption systems, from biomass to nanomaterials, from adsorbents and heavy metals to pharmaceuticals, and from adsorbates to contaminants, as they best fit the experimental data.^11^ The pseudo-second-order model had better correlation coefficient values than the first-order kinetics (Fig. 6c and S9,† and Table 1). The dominant mechanism could be chemical adsorption.^38^

**Table 1:** Rate constants of the pseudo-first-order and pseudo-second-order kinetic models for Pb(ii) adsorption on B–CDs

Pseudo-second order			Langmuir constants			Freundlich constants		
*q*_e_ (mg g^−1^)	*k*_2_ (g mg^−1^ min^−1^)	*R* ^2^	*K*_L_ (L mg^−1^)	*q*_m_ (mg g^−1^)	*R* ^2^	*K*_F_ (mg L^−1^)^1/*n*^	*n*	*R* ^2^
14.2	1.18	0.99	0.03	400	0.99	1.28	1.01	0.98

To this end, the Langmuir and Freundlich isotherms (eqn (5) and (6)) were used. The Langmuir model was better fitted than the Freundlich model based on the correlation coefficients (*R*^2^) from Table 1 and Fig. 6a and b. The maximum adsorption capacity was equal to 400 mg g^−1^ toward Pb(ii) removal, at room temperature and pH = 8 and under light conditions.

### Mechanism

**Fig. 7:**
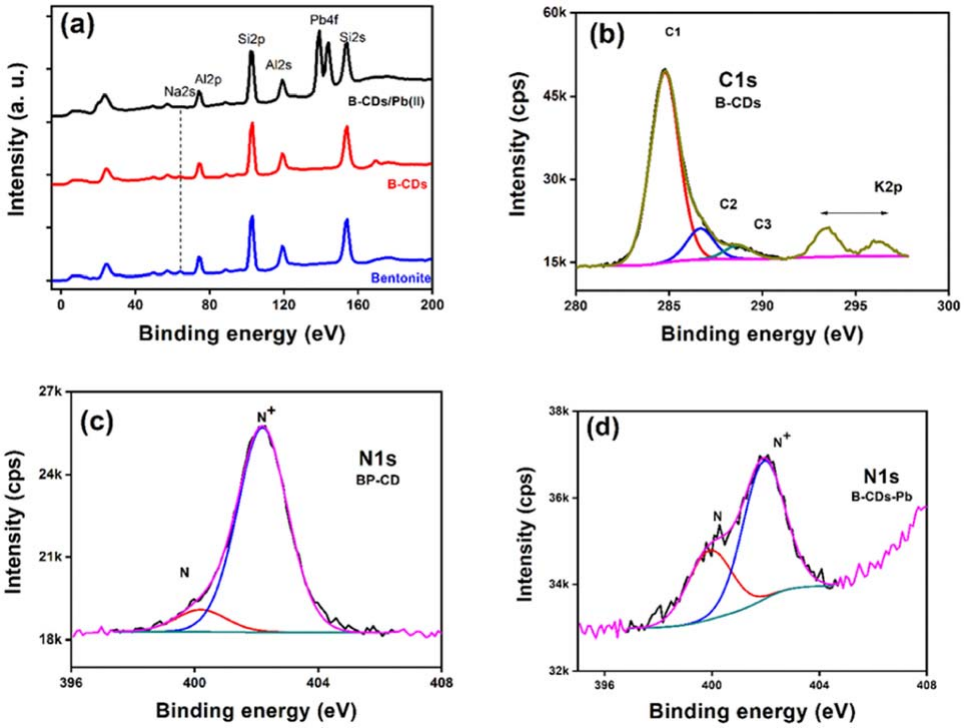
XPS spectra of clay-based materials: (a) survey regions from B, B–CDs, and B–CDs–Pb in the 0–200 eV spectral range; (b) fitted C 1s and K 2p doublet from B–CDs, (c) fitted N 1s region from B–CDs, and (d) fitted N 1s spectrum from B–CDs–Pb. The increasing baseline in (d), starting at 404 eV, is due to Pb 4d_5/2_.

To further confirm the adsorption mechanism of Pb(ii) removal, XPS was performed for the B–CDs materials before and after adsorption, and the XP survey spectra and narrow regions are displayed in Fig. 7. The survey region limited to 0–200 eV (Fig. 7a) permits unambiguous detection and comparison of the peak intensity ratios of the characteristic aluminosilicate specific peaks (Na 2s, Al 2p, and Si 2p) and Pb 4f doublet from the captured lead ions (Pb(ii)). After lead uptake, the spectra were recorded for pristine bentonite, B–CDs, and B–CDs/Pb. One can note that the Al 2p/Si 2p peak height ratio remains the same, which qualitatively indicates that the aluminosilicate composition remains the same (this point will be discussed further). Fig. 7b displays the peak fitted C 1s narrow region from B–CDs. We display this peak for simplicity, as all spectra have similar shapes. C1, C2, and C3 peak components refer to C–C/C–H, C–N/C–O, and O=C–O bonds or functional groups. The N 1s regions are displayed in Fig. 7c (B–CDs) and 7d (B–CDs/Pb); they are fitted with two components centered at 400.1 and 402.1 eV, assigned to free (–NH_2_) and quaternized amine (–NH_3_^+^) groups, respectively.

Table 2 reports the surface chemical composition of bentonite clay before and after intercalation with CDs and the further uptake of Pb(ii) ions. To check for any cation exchange process, the (Na + K)/(Si + Al) atomic ratios can be calculated from Table 2. The Si/Al atomic ratio = 2.10, 2.30, and 2.34 for B, B–CD, and B–CD–Pb; it remains the same (within an acceptable 10% error in XPS), indicating the aluminosilicate backbone's stability. The ratio equals 0.075, 0.048, and 0.038 for B, B–CD, and B–CD–Pb. CDs likely intercalate clay by an ion-exchange reaction due to the quaternized amine group brought by carbon dots. The ratio further decreases upon the uptake of Pb(ii). The N^+^/N ratio drops from 9 to 1.9 upon Pb(ii) uptake. Pb(ii) possibly interacts with COO– groups from carbon dots. For this reason, fewer quaternized nitrogen atoms are detected. The Pb 4f_7/2_ peak is centered at 138.8 eV, less than the value reported for PbSO_4_, the salt employed for the removal experiments. This shows clearly that Pb(ii) is interacting with B–CD. Elsewhere, the Pb 4f_7/2_ peak is reported to be centered at 138.2 eV in the electrosorption of Pb(ii) using graphene/nickel foam electrode material.^36^ In contrast, in the case of lead-silicate glass, the peak of Pb(ii), possibly in the Si–O–Pb chemical environment, is centered at 138.8 eV.^37^ The binding energy of Pb 4f_7/2_ in lead acetate is 138.5 eV.^38^ In addition to the mechanisms mentioned above, physical adsorption was also considered typical for clay-based materials.^39^

**Table 2:** Surface chemical composition of clay-based materials B, B–CDs, and B–CDs after Pb(ii) uptake

Materials	Si	Al	O	Na	K	C1	C2	C3	N	N^+^	S	Pb
B	18.4	8.67	60.5	1.54	0.49	8.36	1.33	0.49	—	—	—	—
B–CDs	17.4	7.55	59.0	0.79	0.4	10.3	1.58	0.69	0.17	1.53	0.68	—
B–CDs–Pb	17.0	7.27	57.9	0.36	0.38	11.4	2.41	1.51	0.26	0.5	—	0.98

Electrostatic attraction may occur between carboxylic groups and Cd(ii);^16^ S and N atoms could donate their electrons to form coordination complexes or covalent bonds with Cd(ii).^40^

**Fig. 8:**
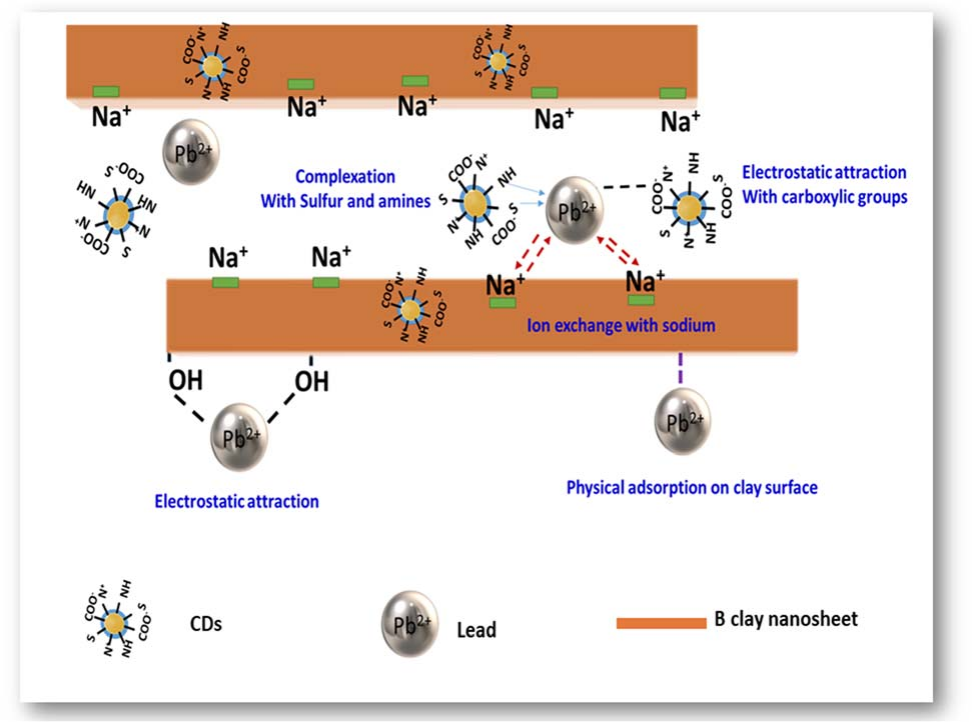
Proposed mechanism of Pb(ii) removal by B–CDs.

Based on the XPS results and explanations mentioned above, a potential adsorption mechanism of Pb(ii) using B–CDs material has been proposed (Fig. 8), which is summarized as follows: (1) complexation of Pb(ii) and N and S groups; (2) electrostatic attraction between Pb(ii) and carboxylic, –NH and –OH groups; (3) ion-exchange between Na^+^ and Pb(ii); (4) physical adsorption of Pb(ii) on the surface of the bentonite clay material.^41^

To conclude, in this work, the mechanism of heavy metal removal is indeed complicated, and this could have arisen from bentonite, as previously reported;^42^ it could be concluded that there is a decrease in Pb(ii) concentration in the presence of the pristine sample (pure clay) (Fig. 6d) and extraction efficiency has reached 80%. This happened because of the cationic exchange between the free monovalent and divalent cation groups in the clay and the Pb(ii).^2^ We can see that from XPS, the sodium atomic ratio decreases from 1.5 to 0.3% after Pb(ii) capture, and a synergetic effect toward heavy metal removal was observed in the presence of well-dispersed N and S co-doped CDs, especially in alkaline solution and under light conditions, which exacerbates their chelating properties. Other previous studies have shown that different adsorption types may represent the interaction between the metallic ions and the used hybrid B–CDs materials, *e.g.*, physical, chemical, or chemi-physisorption.^43,44^ In this work and from the Langmuir isotherm results (Table 2), *K* constants were used to calculate the values of the standard Gibbs free energy change of adsorption (Δ*G*°) according to the following equation (eqn (7))^45^

7 Δ*G*° = −*RT* ln(55.5*K*)

where *R* is the universal gas constant in J mol^−1^ K^−1^, *T* is the temperature in K, and 55.5 is the number of moles of water per liter.^46^ In this work, the calculated Δ*G*° equals −37.401 kJ mol^−1^. Generally, if Δ*G*° ≤ −20 kJ mol^−1^, it is linked to physisorption; if it is ≥−40 kJ mol^−1^, it is linked to chemisorption. In recent reports, Δ*G*° values in the range from −28 to −38 kJ mol^−1^ are interpreted as mixed adsorption (*i.e.*, both physisorption and chemisorption),^47^ which is the case in our study. Moreover, recent studies show that N, O, and S dopants on the carbon dot surface increase the negative charge density of the carbon surface and then enhance the Cd(ii) removal ability compared to the non-doped carbon materials.^16^ Different mechanisms have been suggested to explain the interactions of heavy metals with carbon materials doped with oxygen, nitrogen, and sulfur. Based on Pearson's theory, the affinity of heavy metals towards nitrogen/sulfur is explained by soft acid–soft base interactions, where the B–CDs act as the soft base and Pb(ii) as the soft acid.^48^

In sum, one can say that the mechanism of hybrid Pb(ii) removal using the B–CDs is mixed adsorption, and the removal may have arisen from bentonite or from CDs, separately, or from the hybrid B–CDs, and it can be summarized as follows:

(1) Mixed adsorption on the surface of the B–CDs prepared material (as per the Δ*G*° calculation).^47^

(2) Coordination of Pb(ii) *via* C, O, N, and S groups (present in the carbon dots).^40^

(3) Electrostatic attraction between Pb(ii) and carboxylic, –NH, and –OH groups (present in both carbon dots and clay material).^49^

(4) Cation-exchange between monovalent and bivalent cations present in bentonite clay and Pb(ii).^50^

### Comparison with other adsorbents

Table S2† compares the adsorption capacity of Pb(ii) using the B–CDs nanohybrid with similar materials previously investigated.

As shown in Table S2,† the B–CDs nanohybrid presents superior adsorption capacity compared to other adsorbents due to the formation of a substantial complex between the free carboxylic, N, and sulfur groups offered in CDs and Pb(ii) ions from one side,^51,52^ and the cation exchange reaction from the other side, which may happen between the Na^+^ present in the bentonite clay matrix and the Pb(ii) metal present in solution.^53,54^

## Conclusions

In conclusion, B–CDs were successfully prepared *via* a hydrothermal process using CDs from graphitic waste; natural bentonite served as the host matrix. The CDs were successfully introduced into the bentonite nanoclay matrix and formed well-dispersed B–CDs that were stable and fluorescent. The latter was used as a platform for lead removal from water, with a removal efficiency of around 95%. The adsorption equilibrium time was faster than in other reported studies (only 10 min). Further, the as-prepared B–CDs were fluorescent and exhibited wavelength-dependent photoluminescence properties. The adsorption process could be described using pseudo-second-order kinetics and Langmuir isotherm models; the maximum adsorption capacity was 400 mg g^−1^ toward Pb(ii) removal, at room temperature and pH = 8, under light conditions. This new fluorescent, long-term stable nanohybrid could thus qualify as a low-cost, sustainable, and green adsorbent for the clean water treatment and production sector.

## Conflicts of interest

The authors declare no conflict of interest.

## Supplementary Material

NA-005-D3NA00334E-s001
