# Supplementary material for: A carbon dot-based clay nanocomposite for efficient heavy metal removal
Source: Nanoscale Adv. 2023 Jul 3;5(16):4224–32. doi: 10.1039/d3na00334e (PMC10408590; doi:10.1039/d3na00334e)
Supplement: NA-005-D3NA00334E-s001 [file NA-005-D3NA00334E-s001.pdf]

Electronic Supplementary Material (ESI) for Nanoscale Advances.
This journal is © The Royal Society of Chemistry 2023

Supporting Information

Carbon dots-based clay nanocomposite for efficient heavy metal removal

Khouloud Jlassi[1*], Maryam Al Ejji [1], Abdelgalil Khalaf Ahmed [2], Hafsa Mutahir[2], Mostafa H. Sliem[1,] Aboubakr M. Abdullah[1*], Mohamed M. Chehimi[3, *], Igor Krupa[1].

1. Center for Advanced Materials, Qatar University, P.O. Box 2713, Doha, Qatar
2. College of Arts and Sciences, Qatar University, P.O. Box 2713, Doha, Qatar
3. Université de Paris, ITODYS, UMR CNRS 7086, 15 rue JA de Baïf, 75013 Paris, France

*Correspondence:

khouloud.jlassi@qu.edu.qa (KJ); abubakr2@yahoo.com (AMA); and mohamed.chehimi@cnrs.fr (MMC)

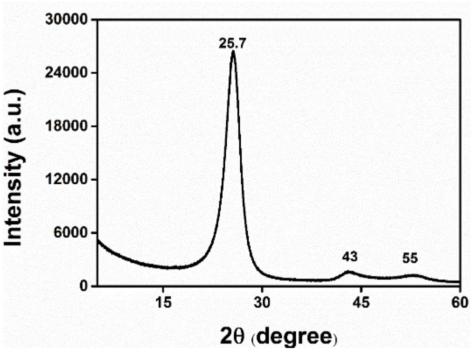

Fig. S1. X-ray diffraction pattern of the graphitic waste

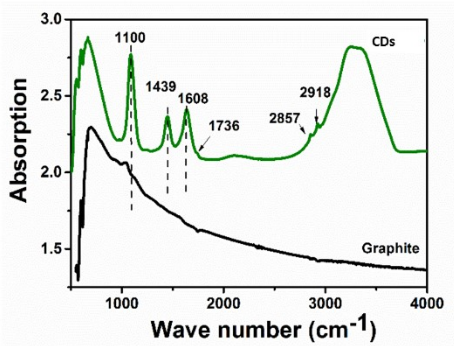

Fig. S2. FTIR of the graphitic waste and resulted in CDs.

Table S1.Calculation of Quantum Yield

	Integrated P.L. intensity	Refractive index	Quantum yield

Quinine sulphate	403862.308	1.33	54%
CDs	2.14755 E-5	1.33	28.7 %

$\Phi C = 54 \times (2.14755\ E\text{-}5/403862.308) \times (1.332/1.332)\ \% = 28.7\ \%$

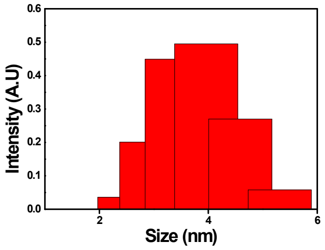

Fig S3.particle size distribution of CDs.

The selectivity test was evaluated in the presence of Cu^{2+}, Zn^{2+}, and Co^{2+} at pH 8.0, and the relative removal efficiency equals 48, 33, 66, and 95 %, respectively (Fig. S4).

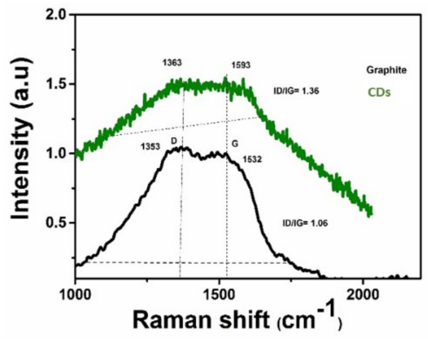

Figure. S4. Raman spectra of graphite waste and CDs derivate.

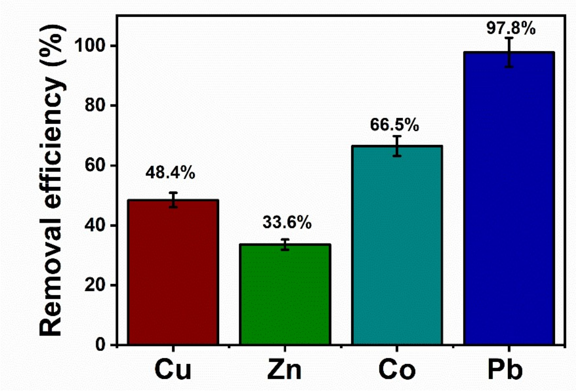

Fig.S5. Selectivity test of B-CDs in presence of 30 ppm of Cu^{2+}, Zn^{2+}, and Co^{2+} at pH 8.0 and light conditions

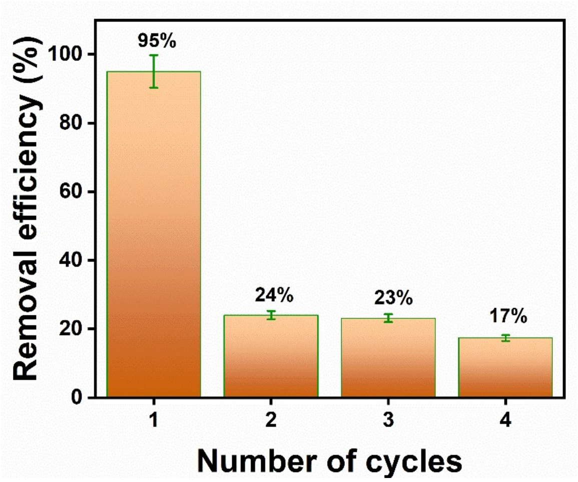

Fig.S6.Effect of regeneration cycles on the removal efficiency of Pb(II) onto B-CDs using HNO_3 as desorbing agent

Chemical stability

To check the chemical stability of B-CDs adsorbent, the latter was dipped in different pH solutions, and the collected dried adsorbents (B-CDs-Pb), were found to be chemically stable, as shown in Fig. S6.

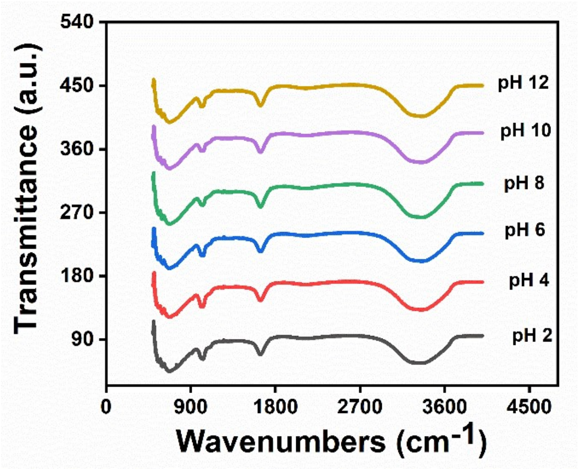

Fig.S7. FTIR spectra of BP-CDs-Pb under different acidic and alkaline conditions

To check the thermal stability of (B-CDs), the Pb removal study was conducted under different temperature, and the FTIR of the collected dried B-CDs-Pb adsorbent show that the used material is thermally stable, as shown in Fig. S7.

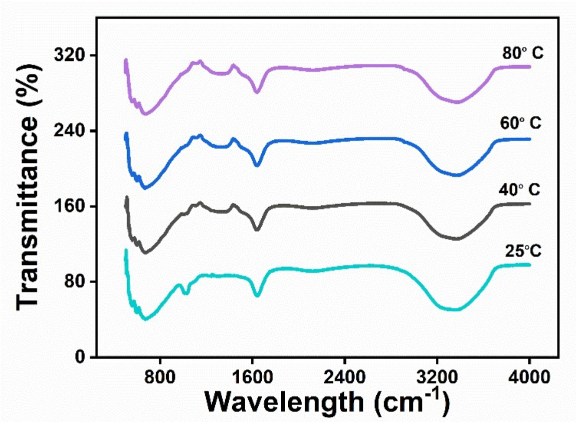

Fig.S8. FTIR spectra of BP-CDs after Pb removal at different under different temperature

First-order and pseudo-second-order models (equations 3, 4) were used to study the variations in adsorption with time[2, 3]. The better correlation coefficient values were noted with the pseudo-second-order model compared to the first-order kinetics (Fig. 5c, S8 and Table 2).

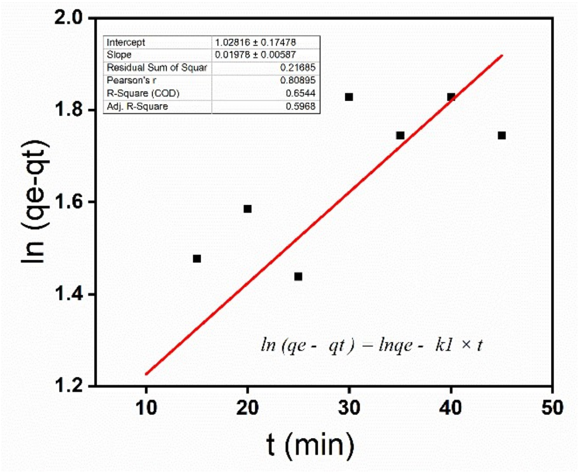

Fig. S9. Pseudo-first -order mode for Pb(II) adsorption using B-CDs under light conditions.

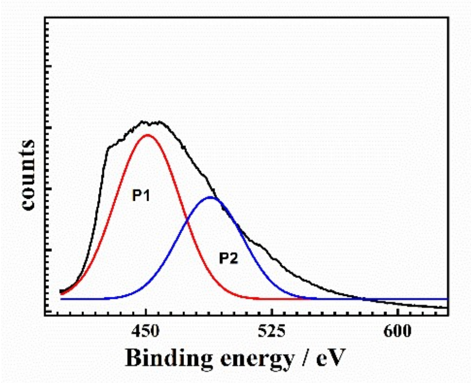

Fig S10. Fitted PL spectrum of the nanoparticles at a 380 nm excitation wavelength.

Comparison with other adsorbents

Table S2 compares the adsorption capacity of Pb(II) using B-CDs nanohybrid with similar materials previously investigated.

Table S2. Comparison of some adsorbent-based clay materials used for Pb(II) removal

**Adsorbent**	**Qm /mg g^{-1}**	**Experimental conditions**	**t t/min**	**References**
Kaolinite clay modified with polyphosphate	40	pH = 5, 25°C	60	1
Bentonite clay	26.3	pH = 4, T = 25 °C	60	2
Diazonium-based ion-imprinted polymer/clay nanocomposite	301	pH = 7, T = 30 °C	30	3
Magnetic Sepiolite Clay	96.15	pH = 7, T = 25 °C	30	4
Attapulgite clay modified with MgO	127.6	pH = 5, T = 55 °C	1440	5
Bentonite modified with Carbon dots	400	pH=8 T = 25°C UV=365 nm	45	**This work**

References:

1. Amer, M. W.; Khalili, F. I.; Awwad, A. M. Journal of environmental chemistry and ecotoxicology **2010,** 2, (1), 001-008.
2. Khan, M.; Hegde, R.; Shabiimam, M. International Journal of Scientific Research and Management **2017,** 5, (7), 5800-5804.
3. Msaadi, R.; Ammar, S.; Chehimi, M. M.; Yagci, Y. European Polymer Journal **2017,** 89, 367-380.
4. Fayazi, M. Analytical and Bioanalytical Chemistry Research **2019,** 6, (1), 125-136.
5. Huang, R.; Lin, Q.; Zhong, Q.; Zhang, X.; Wen, X.; Luo, H. Arabian Journal of Chemistry **2020,** 13, (4), 4994-5008.
